# Supplementary material for: School health professionals’ understanding of culture: a scoping review protocol
Source: BMJ Open. 2023 Nov 24;13(11):e077134. doi: 10.1136/bmjopen-2023-077134 (PMC10680005; doi:10.1136/bmjopen-2023-077134)
Supplement: Author's manuscript [file bmjopen-2023-077134.draft_revisions.pdf]

BMJ Open is committed to open peer review. As part of this commitment we make the peer review history of every article we publish publicly available.

When an article is published we post the peer reviewers' comments and the authors' responses online. We also post the versions of the paper that were used during peer review. These are the versions that the peer review comments apply to.

The versions of the paper that follow are the versions that were submitted during the peer review process. They are not the versions of record or the final published versions. They should not be cited or distributed as the published version of this manuscript.

BMJ Open is an open access journal and the full, final, typeset and author-corrected version of record of the manuscript is available on our site with no access controls, subscription charges or pay-per-view fees (http://bmjopen.bmj.com).

If you have any questions on BMJ Open's open peer review process please email info.bmjopen@bmj.com

BMJ Open

School nurses' and school social workers' understanding of culture: a scoping review protocol.

Journal:	*BMJ Open*
Manuscript ID	bmjopen-2023-077134
Article Type:	Protocol
Date Submitted by the Author:	26-Jun-2023

Complete List of Authors:	Wahlström, Emmie; Mälardalen University, ChiP research group; Mälardalen University, School of health, care and social welfare<br>Landerdahl Stridsberg, Sara; Mälardalen University, University library<br>Larsson, Camilla; Mälardalen University, University library<br>Stier, Jonas; Mälardalen University, School of health, care and social welfare

Keywords:	Schools, Adolescent, PUBLIC HEALTH, Primary Health Care, Health Equity, Community child health < PAEDIATRICS

SCHOLARONE™
Manuscripts

Title:

School nurses' and school social workers' understanding of culture: a scoping review protocol.

Authors:

Emmie WAHLSTRÖM PhD[a,b], Sara LANDERDAHL STRIDSBERG[c], Camilla LARSSON[c], and Jonas STIER Prof., PhD[b]

a. ChiP research group, School of health, care and social welfare, Mälardalen University, Västerås, Sweden
b. School of health, care and social welfare, Mälardalen University, Västerås, Sweden
c. University library, Mälardalen University, Västerås, Sweden.

Corresponding author:

Emmie Wahlström

E-mail address: emmie.wahlstrom@mdu.se

Telephone number: +46 21 101518

Contact details: Mälardalen University, School of health, care and social welfare, Box 883, 721 23 Västerås, Sweden

For peer review only

Abstract:

Introduction: Culture is highlighted in previous research as important in encounters where health professionals and children do not share a language or culture. In these encounters, culture is described as mainly related to the child whereas the health professionals' understanding of their own culture as impacting the encounter tends to be left out. To clarify how culture is understood and conceptualized among professionals, it is of relevance to collate previous research on health professionals' understanding of culture. In the scoping review that this protocol describes, we aim to focus on the context of the school health services, being a context accessible to many children in their everyday life. The aim of the review will be to identify, describe, and analyze previous research concerning school nurses' and school social workers' understanding of culture. It will also highlight gaps in knowledge related to school nurses' and school social workers' understanding of culture.

Methods and Analysis: This scoping review will be guided by the methodology described by Peters et al. and Khalil et al. Searches will be conducted in Scopus, PubMed, Cinahl Plus, SocIndex, Sociological abstracts, Social services abstracts, APA PsycInfo, APA PsycArticles, Web of science and ASSIA. Any published scientific papers focusing on school nurses' or school social workers' understanding of culture (conceptualized through a variety of related terms) and school health services conducted within the last ten years (2013-2023) will be included. Two reviewers will independently screen all titles and abstracts for inclusion. One reviewer will conduct the screening of full-text documents and the extraction of information, which will be validated by the other reviewer. Qualitative content analysis as well as discourse analysis will be employed.

Ethics and dissemination: The findings will be disseminated through peer review publication as well as presentation at conferences and to relevant stakeholders.

Keywords: School nurse, School social worker, culture, children's health, Scoping review protocol

Strengths and limitations of this study:

- This scoping review will provide an overview of research studying school nurses' and school social workers' understanding of culture.
- The methodological description of scoping reviews by Peters et al. and Khalil et al. has been used to plan the review.
- The searches will be conducted in ten electronic databases to identify relevant studies published during the last ten years (2013-2023).
- The review will not include a formal assessment of study quality, as it is a scoping review.

For peer review only

Introduction:

The World Health Organization (WHO, 2022) states that culture is an important factor in the service delivery of refugee- and migrant-sensitive health systems. What does this statement entail? To what does culture refer and how can it be conceptualized?

In political and scientific discourse alike, culture is alternatively conceptualized as an individual background variable, a cognitive scheme, a discursive construction, or as a contextual characteristic. In encounters between migrant children and professionals working within health services, culture can be understood in all these conceptions. There is ample research recognizing the importance of culture in such interaction (Ladha & Neiterman, 2021; Radjack et al., 2020; Zambri et al., 2020), yet in the health and welfare domain the focus is primarily on culture as a background variable or cognitive scheme. Additionally, culture is sometimes highlighted in relation to challenges such as when the professionals and children *lack* a joint culture and or common language (Zambri et al., 2020). By the same token, professionals tend to view culture mainly as a *characteristic of the child* in the encounter whereas the professionals are conceived to be "acultural" (Radjack et al., 2020), or culture is considered as a background variable explaining various health outcomes and healthcare needs among the children (Olcon & Gulbas, 2018).

Moreover, the culture of a child and their family is also stressed as something to acknowledge in encounters. It is also stressed that the cultural needs of children and families should be addressed when providing care (Ladha & Neiterman, 2021). Such "cultural needs" encompass aspects related to, for instance, ethnic traditions, cultural norms and values, or religious beliefs impacting on interaction in general and the provision of healthcare in particular. "Cultural needs" also include aspects related to limited resources available in low-income or rural areas, as well as language and communication "barriers". In relation to the "cultural needs" of children, findings in the same review of healthcare for pediatric patients show that the education of healthcare professionals tends to be ethnocentric, Eurocentric and without cultural self-reflection, i.e., what Stier (2010) describes as discursive blind spots or biases. Thus, rather than counteracting "problems" or misunderstandings, such education biases may fuel constructions of other needs as "different" leaving healthcare professionals without competence to encounter children with needs, values and cultures outside "the norm" (see also Kagawa Singer et al., 2016).

Against this background, there is a need for research addressing culture as a discourse, social construction and interactional accomplishment as well as a need to collate research on the culture(s) within the healthcare services targeting children's health and well-being beyond the pediatric context. One such healthcare service devoted to promoting health and preventing disease among children is the school health services (SHS). In an international context, SHS is multiprofessional, although the most common profession is school nurses (Baltag et al., 2015). Among other professions, it includes doctors, psychologists and social workers. In Sweden, SHS consists mainly of school nurses, school doctors/physicians, school social workers, school psychologists, and special education teachers. Out of these, school nurses are the largest number of professionals working solely within SHS (2 910 persons year 2021; SKR, 2021) followed by school social workers (2 830 persons year 2021; SKR, 2021). These two professional groups often have an office at the school and are available for spontaneous drop-in meetings where the children can discuss issues relating to their health and or well-being. Although the work content of each profession contains aspects unique to the Nordic and Swedish context, similarities have been found with professionals' work internationally (Baltag et al., 2015; Michaud et al., 2021; Beck & Hämäläinen 2022). School nurses

worldwide encounter children where the focus of the encounters is the children's health and screening for health and development deviations (Baltag et al., 2015). Similarly, school social workers worldwide encounter children facing similar problems, e.g., physical and psychological health issues, poverty, issues concerning attendance, dropout and motivation as well as decaying neighborhoods and racism (Beck & Hämäläinen 2022). By being available in the school setting, these professionals can assist children with issues related to health and development in the children's everyday life, – making it interesting to scientifically investigate these professionals' understanding of culture when interacting with children.

Previous studies have been conducted studying school nurses' and school social workers' encounters with children, and have highlighted culture as relevant, especially in encounters with children who have migrated (e.g., Musliu et al., 2019; Inkeroinen et al., 2020; Villarreal Sosa et al., 2021; Wahlström et al., 2021) and in relation to race or ethnicity (e.g., Villarreal Sosa & Martin, 2021). Yet there is a lack of reviews collating this research. A preliminary search for existing scoping reviews within this area of research has been conducted by the research group (SLS and CL) in the Cochrane Library, Epistemonikos, Internationa HTA database, Open Science Framework, Social Care Online and PROSPERO. The searches confirmed that no scoping reviews are or have been conducted previously on this topic.

Objective:

The aim of this review is to identify, describe, and analyze previous research concerning school nurses' and school social workers' understanding of culture. It will also highlight gaps in knowledge related to school nurses' and school social workers' understanding of culture.

Review questions:

- What research has been conducted on school nurses' understanding of culture?
- What research has been conducted on school social workers' understanding of culture?
- In what geographical contexts (i.e., countries) have research concerning school nurses' and school social workers' understanding of culture been conducted?
- How has culture been conceptualized in research concerning school nurses' and school social workers' understanding of culture?
- What knowledge gaps can be found concerning school nurses' and school social workers' understanding of culture?

Methods:

This scoping review protocol follows the structure described by Peters et al. (2022) and the JBI manual (Peters et al., 2020). The methodology described by Peters et al. (2022) and Khalil et al. (2021) has guided the design of the scoping review and how it will be conducted. The scoping review protocol has been planned and designed in collaboration with research librarians (co-authors CL & SLS), who will also conduct the searches for the scoping review.

For peer review only

Eligibility criteria (participants, concepts, context and types of evidence source):

Eligibility criteria for this scoping review are based on the format suggested by Peters et al. (2022) and the JBI manual (Peters et al., 2020) focusing on defining the participants, the concept and the context. In this scoping review, research concerning the two *types of participants* included in the title and aim will be included, e.g., school nurses and school social workers. The profession of school nurse has the same "title" of the profession regardless of geographical context, although the content of what they do might differ somewhat (Baltag et al., 2015; Michaud et al., 2021). However, for the profession of school social worker, the "title" of the profession varies between geographical contexts (Beck & Hämäläinen 2022). To account for these variations, the variation of "titles" described in Beck & Hämäläinen (2022) will be included in the searches. In addition, the descriptions of the profession in the articles generated from the searches will be checked in relation to descriptions of the profession of "school social worker". As counseling is part of the school social workers' practice search, terms related to school counseling will be included, although the profession of school counselor is not included in the scoping review.

The *concept* in focus in the scoping review is culture and in particular professionals' understanding of culture. Therefore, eligible articles for this review are those that explicitly mention culture in the text and that study the professionals' understanding of culture. However, no particular definition of culture will guide the inclusion of articles as one of the research questions concerns how culture has been conceptualized in the studies. Eligible articles are articles that are located in the *context* of SHS. As school systems vary between countries, eligible articles will be articles that include school nurses and or school social workers working in schools where children and adolescents aged 6-19 years of age attend. This implies that articles including professionals working in schools that enroll students at university level (or equivalent level) are not included in the review. The *types of evidence sources* used for this scoping review are published scientific papers, including both reviews and primary studies. To avoid duplicate data, primary studies will be excluded if their results have been included and reported in the results of an included review. An additional limitation of the searches is that the publication date should be within the last 10 years (2013-2023) to reflect the contemporary research field.

Table 1. Search terms used based on the eligibility criteria

**Eligibility criteria**	**Search theme**	**Search terms used**
Participants	School nurses	"school nurs*"
	School social workers	"school social work*", "school welfare officer*", "education social worker", "education welfare officer"*, "attendance counselor", "school counsel*", "guidance counselor*"
Concept	Understanding of culture	"cultural* competen*", "cultural* sensitiv*", transcultural, "cross cultural", "cultural* congruent*", "cultural care", multicultur*, "cultural divers*", intercultural, "cultural awareness", "cultural nursing", "cultural communication", "indigenous knowledge", "cultural skill*", "cultural understanding", "cultural

For peer review only

		interaction*", "cultural knowledge", "cultural proficienc*", "cultural dynamic*", "cultural safety", "cultural meeting"
Context	School health services	"school health", "school based health", "school mental health service*", "school based mental health service*",

Search strategy:

As studies of school nurses and school social workers might be published in journals with varying scopes, from medicine to psychology and social work, a wide range of databases will be included. Searches will be conducted in Scopus(https://www.scopus.com/), PubMed(https://pubmed.ncbi.nlm.nih.gov/), Cinahl Plus(EBSCOhost), SocIndex(EBSCOhost), Sociological abstracts(ProQuest), Social services abstracts(ProQuest), APA PsycInfo(EBSCOhost), APA PsycArticles(EBSCOhost), Web of Science (https://www.webofscience.com) and ASSIA(ProQuest). The search terms (see Table 1) will be combined using the Boolean operators AND and OR, and phrase searching and truncation will be applied where appropriate. The search strategy will include a combination of free text terms and, where applicable, controlled vocabulary which relates to the eligibility criteria of the review (participants, concept and context). The full initial search strategy is provided in Table 2.

In accordance with Peters et al. (2022), the searches may be modified and expanded during the process to account for any new potentially relevant terms, concepts or contexts based on the results of searches or the process of evidence selection. Conducted searches will also be accompanied by manual searches of relevant references cited in the included studies, in the Web of Science Core Collection, Scopus and Google Scholar.

Table 2. Initial search strategy

**Blocks**	**Search string / search terms**
1.	TITLE-ABS-KEY ("school health" OR "school based health" OR "school nurs*" OR "school mental health service*" OR "school based mental health service*" OR "school social work*" OR "school welfare officer*" OR "education social worker" OR "education welfare officer*" OR "attendance counselor" OR "school counsel*" OR "guidance counselor*")
2.	TITLE-ABS-KEY ("cultural* competen*" OR "cultural* sensitiv*" OR transcultural OR "cross cultural" OR "cultural* congruent*" OR "cultural care" OR multicultur* OR "cultural divers*" OR intercultural OR "cultural awareness" OR "cultural nursing" OR "cultural communication" OR "indigenous knowledge" OR "cultural skill*" OR "cultural understanding" OR "cultural interaction*" OR "cultural knowledge" OR "cultural proficienc*" OR "cultural dynamic*" OR "cultural safety" OR "cultural meeting")
**Combination**	1 AND 2
**Limitations**	2013-2023

For peer review only - http://bmjopen.bmj.com/site/about/guidelines.xhtml

Study/source of evidence selection:

To assist in the evidence selection process and as a tool for deduplication, the Covidence software will be used. The selection of sources will begin by screening the title and abstract of each publication in the search results. Initial screening will be conducted by EW and JS for titles and abstracts against the inclusion criteria. The inclusion of articles will be guided by the population, concept and context as well as types of evidence described above in the eligibility criteria. This process will also assist in the clarification of inclusion and exclusion criteria if needed. Agreement in the selection process will be determined through discussions between authors regarding the relevance of selected publications to the eligibility criteria until negotiated consensus is reached. Thereafter, the included full-text publications will be examined. EW will conduct the full-text screening and JS will evaluate a random sample of 10% of full-text articles to validate inclusion decisions.

Exclusion of publications will be shown using a flow diagram or chart such as the adapted chart in the PRISMA-ScR. Studies will be excluded if full-text documents cannot be retrieved. Excluded publications and reasons for exclusion will also be reported in an appendix for all publications examined in full text.

Data extraction:

Data from the included full-text publications will be processed using the Covidence software and exported to a Microsoft Excel spreadsheet containing the following headings: Author(s), Year of publication, where the source was published or conducted (country of origin), aims/purpose, population and sample size, methodology/methods, outcomes and details of these (if applicable), conceptualization of culture as well as key findings that relate to the scoping review questions. If needed during the charting process, the data extraction spreadsheet might be updated to include additional headings to better capture content relevant to the scoping review. Extraction will be conducted by EW and validated by JS. Discrepancies in extraction will be discussed until negotiated consensus is reached.

Data analysis and presentation:

Data analysis will consist of summarizing the extracted data related to methodology, professions, and geographical contexts to answer related research questions. In addition, extracted data related to the conceptualization of culture and key findings will be analyzed using qualitative content analysis and discourse analysis. The results will be described in relation to the research questions and as appropriate in relation to each analysis methodology. The results of the discourse analysis of the included publications will be presented in a separate publication.

Conclusion:

This scoping review aims to identify, describe, and analyze the content in previous research concerning school nurses' and school social workers' understanding of culture. It will also highlight gaps in knowledge related to school nurses' and school social workers' understanding of culture. Collating and summarizing current knowledge on these professions' understandings of culture is important for continuing the improvement of the delivery of school health services. The review will be conducted as a first part of a study investigating Swedish school nurses' and school social workers' understanding of culture. The findings of this review will provide guidance for the

For peer review only - http://bmjopen.bmj.com/site/about/guidelines.xhtml

development of this study and will also contribute to the collation of existing research within the field.

Ethics and dissemination

This review does not require ethics approval. The dissemination strategy includes peer review publication and presentation at conferences and to relevant stakeholders.

Author contributions:

EW and JS conceptualized the idea and study design. SLS and CL contributed to discussions on study design, conducted searches of existing literature reviews and conducted initial test searches to clarify search terms and search strategies. EW drafted the first version of the protocol. JS, SLS and CL critically commented and/or revised the text.

Furthermore, all authors have given final approval of the version to be published and agree to be accountable for all aspects of the work.

Funding statement:

This research received no specific grant from any funding agency in the public, commercial or not-for-profit sectors.

Competing interests statement:

The authors declare that they have no competing interests.

Patient and public involvement statement

The scoping review protocol has been developed without any patient or public involvement. This scoping review is not planned to include patient or public involvement.

Word Count: 2 555 words.

For peer review only

References:

Baltag, V., Pachyna, A., & Hall, J. (2015). Global Overview of School Health Services: Data from 102 Countries. *Health Behavior & Policy Review, 2*(4), 268-283. http://dx.doi.org/10.14485/HBPR.2.4.4

Beck, K. F., & Hämäläinen, J. (2022). Mapping the field of international comparative research in school social work. *International Social Work, 65*(2), 203–223. https://doi.org/10.1177/0020872819897742

Inkeroinen, S., Hupli, M., & Stolt, M. (2020). School nurses' experiences of health promotion for school-age asylum seekers. *The journal of school nursing*, Article 1059840520978197. https://doi.org/10.1177/1059840520978197

Kagawa Singer, M., Dressler, W., George, S., & NIH Expert Panel (2016). Culture: The missing link in health research. *Social science & medicine, 170*, 237–246. https://doi.org/10.1016/j.socscimed.2016.07.015

Khalil, H., Peters, M. D., Tricco, A. C., Pollock, D., Alexander, L., McInerney, P., Godfrey, C. M., & Munn, Z. (2021). Conducting high quality scoping reviews-challenges and solutions. *Journal of clinical epidemiology, 130,* 156–160. https://doi.org/10.1016/j.jclinepi.2020.10.009

Ladha, R., & Neiterman, E. (2021). Shades of care: Understanding the needs of racially and ethnically diverse paediatric patients, their families, and health care providers in North America. *Journal of child health care,* Article 13674935211041863. https://doi.org/10.1177/13674935211041863

Michaud, P-A., Vervoort, J. P. M., Visser, A., Baltag, V., Reijneveld, S. A., Kocken, P. L., & Jansen, D. (2021). Organization and activities of school health services among EU countries. *European Journal of Public Health,* 1-6. https://doi.org/10.1093/eurpub/ckaa200

Musliu, E., Vasic, S., Clausson, E. K., & Garmy, P. (2019). School nurses' experiences working with unaccompanied refugee children and adolescents: A qualitative study. *SAGE Open Nursing, 5*, 1-8. https://doi.org/10.1177/2377960819843713

Olcoń, K., & Gulbas, L. E. (2018). "Because That's the Culture": Providers' Perspectives on the Mental Health of Latino Immigrant Youth. *Qualitative Health Research*, *28*(12), 1944–1954. https://doi.org/10.1177/1049732318795674

Peters MDJ, Godfrey C, McInerney P, Munn Z, Tricco AC, Khalil, H. Chapter 11: Scoping Reviews (2020 version). In: Aromataris E, Munn Z (Editors). *JBI Manual for Evidence Synthesis, JBI, 2020*. Available from https://synthesismanual.jbi.global. https://doi.org/10.46658/JBIMES-20-12

Peters, M. D. J., Marnie, C., Tricco, A. C., Pollock, D., Munn, Z., Alexander, L., McInerney, P., Godfrey, C. M., & Khalil, H. (2020). Updated methodological guidance for the conduct of scoping reviews. *JBI evidence synthesis, 18*(10), 2119–2126. https://doi.org/10.11124/JBIES-20-00167

Peters, M. D. J., Godfrey, C., McInerney, P., Khalil, H., Larsen, P., Marnie, C., Pollock, D., Tricco, A. C., & Munn, Z. (2022). Best practice guidance and reporting items for the development of scoping review protocols. *JBI evidence synthesis, 20*(4), 953–968. https://doi.org/10.11124/JBIES-21-00242

Radjack, R., Touhami, F., Woestelandt, L., Minassian, S., Mouchenik, Y., Lacha,l J., & Moro, M.R. (2020). Cultural Competence of Professionals Working With Unaccompanied Minors: Addressing Empathy by a Shared Narrative. *Front. Psychiatry, 11*(528). doi: 10.3389/fpsyt.2020.00528

Stier, J. (2010). The blindspots and biases of intercultural communication studies: A discussion on episteme and doxa in a field. *Intercultural Communication, 24*, 1-9. http://www.immi.se/intercultural/nr24/stier-24.htm

Sveriges kommuner och regioner [SKR] (2021). *Nuläge och utmaningar i elevhälsan 2021*. https://skr.se/skr/tjanster/rapporterochskrifter/publikationer/nulageochutmaningarielevhalsan2021.66776.html

Villarreal Sosa, L., & Martin, M. (2021). Constructions of Race and Equity in a Suburban School: Teachers, School Social Workers, and Other School Staff as Nepantleras and Border Crossers, *Children & Schools*, *43*(1), 19–31. https://doi.org/10.1093/cs/cdaa031

Villarreal Sosa, L., Roth, B., & Rodriguez, S. (2021). Crossing Borders: Exploring the Role of School Social Workers in Immigrant-Serving Schools, *Social Work Research*, *45*(3), 198–206. https://doi.org/10.1093/swr/svab011

Wahlström, E., Golsäter, M., Granlund, M., Holmström, I.K., Larm, P., & Harder, M. (2021) Adjusting and doing the same: School nurses' descriptions of promoting participation in health visits with children of foreign origin. *BMC Public Health, 21*(111):1-11. https://doi.org/10.1186/s12889-020-10144-2

World Health Organization (2022). *World report on the health of refugees and migrants*. https://www.who.int/teams/health-and-migration-programme/world-report-on-the-health-of-refugees-and-migrants

Zambri, F., Marchetti, F., Colaceci, S., Benelli, E., Serra, D., Canevelli, M., Vanacore, N., & Giusti, A. (2020). Taking care of minor migrants' health: The professionals' perception and training needs. *Annali dell'Istituto superiore di sanita, 56*(4), 470–477. https://doi.org/10.4415/ANN_20_04_09

For peer review only

For peer review only

Preferred Reporting Items for Systematic reviews and Meta-Analyses extension for Scoping Reviews (PRISMA-ScR) Checklist

SECTION	ITEM	PRISMA-ScR CHECKLIST ITEM	REPORTED ON PAGE #
**TITLE**			
Title	1	Identify the report as a scoping review.	1
**ABSTRACT**			
Structured summary	2	Provide a structured summary that includes (as applicable): background, objectives, eligibility criteria, sources of evidence, charting methods, results, and conclusions that relate to the review questions and objectives.	1-2
**INTRODUCTION**			
Rationale	3	Describe the rationale for the review in the context of what is already known. Explain why the review questions/objectives lend themselves to a scoping review approach.	3-4
Objectives	4	Provide an explicit statement of the questions and objectives being addressed with reference to their key elements (e.g., population or participants, concepts, and context) or other relevant key elements used to conceptualize the review questions and/or objectives.	4
**METHODS**			
Protocol and registration	5	Indicate whether a review protocol exists; state if and where it can be accessed (e.g., a Web address); and if available, provide registration information, including the registration number.	This is the protocol
Eligibility criteria	6	Specify characteristics of the sources of evidence used as eligibility criteria (e.g., years considered, language, and publication status), and provide a rationale.	5
Information sources*	7	Describe all information sources in the search (e.g., databases with dates of coverage and contact with authors to identify additional sources), as well as the date the most recent search was executed.	6
Search	8	Present the full electronic search strategy for at least 1 database, including any limits used, such that it could be repeated.	6
Selection of sources of evidence†	9	State the process for selecting sources of evidence (i.e., screening and eligibility) included in the scoping review.	7
Data charting process‡	10	Describe the methods of charting data from the included sources of evidence (e.g., calibrated forms or forms that have been tested by the team before their use, and whether data charting was done independently or in duplicate) and any processes for obtaining and confirming data from investigators.	7
Data items	11	List and define all variables for which data were sought and any assumptions and simplifications made.	5-6
Critical appraisal of individual sources of evidence§	12	If done, provide a rationale for conducting a critical appraisal of included sources of evidence; describe the methods used and how this information was used in any data synthesis (if appropriate).	No quality appraisal is conducted

St. Michael's
Inspired Care. Inspiring Science.

For peer review only - http://bmjopen.bmj.com/site/about/guidelines.xhtml

SECTION	ITEM	PRISMA-ScR CHECKLIST ITEM	REPORTED ON PAGE #
Synthesis of results	13	Describe the methods of handling and summarizing the data that were charted.	7
**RESULTS**			
Selection of sources of evidence	14	Give numbers of sources of evidence screened, assessed for eligibility, and included in the review, with reasons for exclusions at each stage, ideally using a flow diagram.	Inapplicable
Characteristics of sources of evidence	15	For each source of evidence, present characteristics for which data were charted and provide the citations.	Inapplicable
Critical appraisal within sources of evidence	16	If done, present data on critical appraisal of included sources of evidence (see item 12).	Inapplicable
Results of individual sources of evidence	17	For each included source of evidence, present the relevant data that were charted that relate to the review questions and objectives.	Inapplicable
Synthesis of results	18	Summarize and/or present the charting results as they relate to the review questions and objectives.	Inapplicable
**DISCUSSION**			
Summary of evidence	19	Summarize the main results (including an overview of concepts, themes, and types of evidence available), link to the review questions and objectives, and consider the relevance to key groups.	Inapplicable
Limitations	20	Discuss the limitations of the scoping review process.	Inapplicable
Conclusions	21	Provide a general interpretation of the results with respect to the review questions and objectives, as well as potential implications and/or next steps.	7
**FUNDING**			
Funding	22	Describe sources of funding for the included sources of evidence, as well as sources of funding for the scoping review. Describe the role of the funders of the scoping review.	8

JBI = Joanna Briggs Institute; PRISMA-ScR = Preferred Reporting Items for Systematic reviews and Meta-Analyses extension for Scoping Reviews.
* Where *sources of evidence* (see second footnote) are compiled from, such as bibliographic databases, social media platforms, and Web sites.
† A more inclusive/heterogeneous term used to account for the different types of evidence or data sources (e.g., quantitative and/or qualitative research, expert opinion, and policy documents) that may be eligible in a scoping review as opposed to only studies. This is not to be confused with *information sources* (see first footnote).
‡ The frameworks by Arksey and O'Malley (6) and Levac and colleagues (7) and the JBI guidance (4, 5) refer to the process of data extraction in a scoping review as data charting.
§ The process of systematically examining research evidence to assess its validity, results, and relevance before using it to inform a decision. This term is used for items 12 and 19 instead of "risk of bias" (which is more applicable to systematic reviews of interventions) to include and acknowledge the various sources of evidence that may be used in a scoping review (e.g., quantitative and/or qualitative research, expert opinion, and policy document).

From: Tricco AC, Lillie E, Zarin W, O'Brien KK, Colquhoun H, Levac D, et al. PRISMA Extension for Scoping Reviews (PRISMAScR): Checklist and Explanation. Ann Intern Med. 2018;169:467–473. doi: 10.7326/M18-0850.

For peer review only - http://bmjopen.bmj.com/site/about/guidelines.xhtml

BMJ Open

School health professionals’ understanding of culture: a scoping review protocol

Journal:	*BMJ Open*
Manuscript ID	bmjopen-2023-077134.R1
Article Type:	Protocol
Date Submitted by the Author:	17-Oct-2023
Complete List of Authors:	Wahlström, Emmie; Mälardalen University, ChiP research group; Mälardalen University, School of health, care and social welfare<br>Landerdahl Stridsberg, Sara; Mälardalen University, University library<br>Larsson, Camilla; Mälardalen University, University library<br>Stier, Jonas; Mälardalen University, School of health, care and social welfare
**Primary Subject Heading**:	Public health
Secondary Subject Heading:	Global health, Sociology
Keywords:	Schools, Adolescent, PUBLIC HEALTH, Primary Health Care, Health Equity, Community child health < PAEDIATRICS

SCHOLARONE™
Manuscripts

Title:

School health professionals' understanding of culture: a scoping review protocol.

Authors:

Emmie WAHLSTRÖM PhD [a, b], Sara LANDERDAHL STRIDSBERG [c],, Camilla LARSSON [c], and Jonas STIER Prof., PhD, [a, b]

a. ChiP research group, School of health, care and social welfare, Mälardalen University, Västerås, Sweden
b. School of health, care and social welfare, Mälardalen University, Västerås, Sweden
c. University library, Mälardalen University, Västerås, Sweden.

Corresponding author:

Emmie Wahlström

E-mail address: emmie.wahlstrom@mdu.se

Telephone number: +46 21 101518

Contact details: Mälardalen University, School of health, care and social welfare, Box 883, 721 23 Västerås, Sweden

Abstract:

Introduction: Culture is highlighted in previous research as important in encounters where health professionals and children do not share a language or culture. In these encounters, culture is described as mainly related to the child whereas the health professionals' understanding of their own culture as impacting the encounter tends to be left out. To clarify how culture is understood and conceptualized among professionals, it is of relevance to collate previous research on health professionals' understanding of culture. In the scoping review that this protocol describes, we aim to focus on the context of the school health services, being a context accessible to many children in their everyday life. The aim of the review will be to identify, describe, and analyze previous research concerning school health professionals' (i.e., school nurses, school social workers, school doctors and school psychologists) understanding of culture.

Methods and Analysis: This scoping review will be guided by the methodology described by Peters et al. and Khalil et al. Searches will be conducted in Scopus, PubMed, Cinahl Plus, SocIndex, Sociological abstracts, Social services abstracts, APA PsycInfo, APA PsycArticles, Web of science and ASSIA. Any published scientific papers focusing on school health professionals' understanding of culture (conceptualized through a variety of related terms) and school health services conducted within the last ten years (2013-2023) will be included. Two reviewers will independently screen all titles and abstracts for inclusion. Two reviewers will conduct the screening of full-text documents and the extraction of information. Qualitative content analysis as well as discourse analysis will be employed.

For peer review only

Ethics and dissemination: Ethical approval is not required for this study. The findings will be disseminated through peer review publication as well as presentation at conferences and to relevant stakeholders.

Keywords: School nurse, School social worker, school doctor, school psychologist, school health professionals, culture, children's health, Scoping review protocol

Strengths and limitations of this study:

- The methodological description of scoping reviews by Peters et al. and Khalil et al. has been used to plan the review.
- The searches will be conducted in ten electronic databases to identify relevant studies published during the last ten years (2013-2023).
- The review will not include a formal assessment of study quality, as it is a scoping review.

For peer review only

Introduction:

Global trends suggest that more people have migrated than ever before. In 2020, about 281 million people were international migrants. [1] Most of these people were in the age of 15 to 64 years and about 15 percent of migrants were below the age of 19. To provide high quality health care services to migrants, the World Health Organization states that culture is an important factor in the service delivery of refugee- and migrant-sensitive health systems. [2] What does this statement entail? To what does culture refer and how can it be conceptualized?

In political and scientific discourse alike, culture is alternatively conceptualized as an individual background variable, a cognitive scheme, a discursive construction, or as a contextual characteristic. In encounters between migrant children and professionals working within health services, culture can be understood in all these conceptions. There is ample research recognizing the importance of culture in such interaction, [3, 4, 5] yet in the health and welfare domain the focus is primarily on culture as a background variable or cognitive scheme. Additionally, culture is sometimes highlighted in relation to challenges such as when the professionals and children *lack* a joint culture and or common language. [5] By the same token, professionals tend to view culture mainly as a *characteristic of the child* in the encounter whereas the professionals are conceived to be "acultural", [4] or culture is considered as a background variable explaining various health outcomes and healthcare needs among the children. [6]

Moreover, the culture of a child and their family is also stressed as something to acknowledge in encounters. It is also stressed that culture of children and families should be considered when providing care and accounting for their needs. [3] Such "cultural considerations" encompass, for instance, ethnic traditions, cultural norms and values, or religious beliefs impacting on interaction in general and the provision of healthcare in particular. "Cultural considerations" also include aspects related to limited resources available in low-income or rural areas, as well as language and communication "barriers". In relation to the "cultural considerations" of children, findings in the same review of healthcare for pediatric patients show that the education of healthcare professionals tends to be ethnocentric, Eurocentric and without cultural self-reflection, i.e., what Stier describes as discursive blind spots or biases. [7] Thus, rather than counteracting "problems" or misunderstandings or merely focusing on the culture of children parents, such biases in education or among professionals, for instance in school health care services, may fuel constructions of other needs as "different" leaving healthcare professionals without competence to encounter children with needs, values and cultures outside "the norm". [8]

Against this background, there is a need for research addressing culture as a discourse, social construction and interactional accomplishment as well as a need to collate research on the culture(s) within the healthcare services targeting children's health and well-being beyond the pediatric context. One such healthcare service devoted to promoting health and preventing disease among children is the school health services (SHS). In an international context, SHS is multiprofessional, although the most common profession is school nurses. [9] Among other professions, it includes doctors, psychologists and social workers. In Sweden, SHS consists mainly of school nurses, school doctors/physicians, school social workers, school psychologists, and special education teachers. Out of these, school nurses are the largest number of professionals working solely within SHS (2 910 persons year 2021) followed by school social workers (2 830 persons year 2021). [10] These two professional groups often have an office at the school and are available for spontaneous drop-in meetings where the children can discuss issues relating to their health and or well-being. School

For peer review only - http://bmjopen.bmj.com/site/about/guidelines.xhtml

doctors and school psychologists mainly serve as consultants, often to several schools within a limited geographical area (municipality or region). Although the work content of each profession contains aspects unique to the Nordic and Swedish context, similarities have been found with professionals' work internationally. [9, 11, 12] School nurses and school doctors worldwide encounter children to assess and screen the children's health and development, vaccinate children against diseases and provide health education. [9] Similarly, school social workers worldwide encounter children facing similar problems, e.g., physical and psychological health issues, poverty, issues concerning attendance, dropout and motivation as well as decaying neighborhoods and racism. [12] Also, school psychologists are available in many countries [13] and work with similar activities. [14] By being available in the school setting, these professionals can assist children with issues related to health and development in the children's everyday life, – making it interesting to scientifically investigate these professionals' understanding of culture when interacting with children.

Previous studies have been conducted studying preschool teachers, teachers, school nurses', school social workers' and school psychologists' encounters with children, and have highlighted culture as relevant, especially in encounters with children who have migrated [15, 16, 17, 18, 19, 20] and in relation to race or ethnicity. [21] Yet there is a lack of reviews collating research regarding school health professionals' (i.e., school nurses, school social workers, school doctors and school psychologists) understanding of culture. A preliminary search for existing scoping reviews within this area of research has been conducted by the research group (SLS and CL) in the Cochrane Library, Epistemonikos, Internationa HTA database, Open Science Framework, Social Care Online and PROSPERO. The searches confirmed that no scoping reviews are or have been conducted previously on this topic.

Objective:

This review aims to identify, describe, and analyze existing research on school health professionals' (i.e., school nurses, school social workers, school doctors and school psychologists) understanding of culture.

Review questions:

- What research has been conducted on school nurses', school social workers', school doctors and school psychologists' understanding of culture?
- What research has been conducted on school nurses', school social workers', school doctors and school psychologists' cultural self-understanding?
- What research has been conducted on intercultural interaction and communication in the context of school health professionals' encounters with students and their families?
- In what geographical contexts (i.e., countries) have research concerning school nurses', school social workers', school doctors and school psychologists' understanding of culture been conducted?
- How has culture been conceptualized in research concerning school nurses', school social workers', school doctors and school psychologists' understanding of culture?

For peer review only

Methods:

Patient and public involvement statement

The scoping review protocol has been developed without any patient or public involvement. This scoping review is not planned to include patient or public involvement.

This scoping review protocol follows the structure described by Peters et al. and the JBI manual. [22, 23] The methodology described by Peters et al. and Khalil et al. has guided the design of the scoping review and how it will be conducted. [22, 24] The scoping review protocol has been planned and designed in collaboration with research librarians (co-authors CL & SLS), who will also conduct the searches for the scoping review.

Eligibility criteria (participants, concepts, context and types of evidence source):

Eligibility criteria for this scoping review are based on the format suggested by Peters et al. and the JBI manual focusing on defining the participants, the concept and the context. [22, 23] In this scoping review, research concerning the four *types of participants* included in the aim will be included, e.g., school nurses, school social workers, school doctors and school psychologists. The profession of school nurse, school doctor or physician and school psychologist has the same "title" of the profession regardless of geographical context, although the content of what they do might differ somewhat. [9, 11, 13, 14] However, for the profession of school social worker, the "title" of the profession varies between geographical contexts. [12] To account for these variations, the variation of "titles" described in Beck & Hämäläinen will be included in the searches. In addition, the descriptions of the profession in the articles generated from the searches will be checked in relation to descriptions of the profession of "school social worker". As counseling is part of the school social workers' practice search, terms related to school counseling will be included, although the profession of school counselor is not included in the scoping review.

The *concept* in focus in the scoping review is culture, more specifically professional's understanding of culture. Therefore, eligible articles for this review are those that mention professional's understanding of culture in the title, abstract, key words or findings. However, no particular definition of culture will guide the inclusion of articles as one of the research questions concerns how culture has been conceptualized in the studies. Eligible articles are articles that are located in the *context* of SHS. As school systems vary between countries, eligible articles will be articles that include school health professionals working at schools or regularly visiting schools where children and adolescents aged 6-19 years of age attend. This implies that articles including school health professionals working in schools that enroll students at university level (or equivalent level) are not included in the review. The *types of evidence sources* used for this scoping review are published scientific papers, including both reviews and primary studies as well as descriptive articles presenting theoretical frameworks, models or concepts. To avoid duplicate data, primary studies will be excluded if their results have been included and reported in the results of an included review. An additional limitation of the searches is that the publication date should be within the last 10 years (2013-2023) to reflect the contemporary research field.

For peer review only - http://bmjopen.bmj.com/site/about/guidelines.xhtml

Table 1. Search terms used based on the eligibility criteria

**Eligibility criteria**	**Search theme**	**Search terms used**
Participants	School nurses	"school nurs*"
	School doctors	"school doctor*", "school physician*"
	School Psychologists	"school psych*"
	School social workers	"school social work*", "school welfare officer*", "education social worker", "education welfare officer"*, "attendance counselor", "school counsel*", "guidance counselor*"
Concept	Understanding of culture	"cultural* competen*", "cultural* sensitiv*", transcultural, "cross cultural", "cultural* congruent*", "cultural care", multicultur*, "cultural divers*", intercultural, "cultural awareness", "cultural nursing", "cultural communication", "indigenous knowledge", "cultural skill*", "cultural understanding", "cultural interaction*", "cultural knowledge", "cultural proficienc*", "cultural dynamic*", "cultural safety", "cultural meeting", "cultural bias", "cultural self*", "cultural identification"
Context	School health services	"school health", "school based health", "school mental health service*", "school based mental health service*",

For peer review only

Search strategy:

As studies of school health professionals might be published in journals with varying scopes, from medicine to psychology and social work, a wide range of databases will be included. Searches will be conducted in Scopus (https://www.scopus.com/), PubMed (https://pubmed.ncbi.nlm.nih.gov/), Cinahl Plus (EBSCOhost), SocIndex (EBSCOhost), Sociological abstracts (ProQuest), Social services abstracts (ProQuest), APA PsycInfo (EBSCOhost), APA PsycArticles (EBSCOhost), Web of Science (https://www.webofscience.com) and ASSIA (ProQuest). The search terms (see Table 1) will be combined using the Boolean operators AND and OR, and phrase searching and truncation will be applied where appropriate. The search strategy will include a combination of free text terms and, where applicable, controlled vocabulary which relates to the eligibility criteria of the review (participants, concept and context). The full initial search strategy is provided in Table 2.

In accordance with Peters et al., [22] the searches may be modified and expanded during the process to account for any new potentially relevant terms, concepts or contexts based on the results of searches or the process of evidence selection. Conducted searches will also be accompanied by manual searches of relevant references cited in the included studies, in the Web of Science Core Collection, Scopus and Google Scholar.

Table 2. Initial search strategy

Blocks	Search string / search terms
1.	TITLE-ABS-KEY ("school health" OR "school based health" OR "school nurs*" OR "school mental health service*" OR "school based mental health service*" OR "school social work*" OR "school welfare officer*" OR "education social worker" OR "education welfare officer*" OR "attendance counselor" OR "school counsel*" OR "guidance counselor*" OR “school doctor” OR “school physician” OR “school psych*”)
2.	TITLE-ABS-KEY ("cultural* competen*" OR "cultural* sensitiv*" OR transcultural OR "cross cultural" OR "cultural* congruent*" OR "cultural care" OR multicultur* OR "cultural divers*" OR intercultural OR "cultural awareness" OR "cultural nursing" OR "cultural communication" OR "indigenous knowledge" OR "cultural skill*" OR "cultural understanding" OR "cultural interaction*" OR "cultural knowledge" OR "cultural proficienc*" OR "cultural dynamic*" OR "cultural safety" OR "cultural meeting" OR “cultural bias” OR “cultural self*”, “cultural identification”)
**Combination**	1 AND 2
**Limitations**	2013-2023

Study/source of evidence selection:

To assist in the evidence selection process and as a tool for deduplication, the Covidence software will be used. The selection of sources will begin by screening the title and abstract of each publication in the search results. Initial screening will be conducted by EW and JS for titles and abstracts against the inclusion criteria. The inclusion of articles will be guided by the population, concept and context as well as types of evidence described above in the eligibility criteria. This process will also assist in the clarification of inclusion and exclusion criteria if needed. Agreement in the selection process will be determined through discussions between authors regarding the relevance of selected publications to the eligibility criteria until negotiated consensus is reached. Thereafter, the included full-text publications will be examined by EW and JS. Calibration of inclusion between reviewers will be conducted by selecting five percent of the articles for screening by both reviewers and control for agreement in decisions at both the intial and the full-text stages. An agreement of 90 percent is strived for and if this is not reached disagreements will be discussed and inclusion criterias revised accordingly. [25]

Exclusion of publications will be shown using a flow diagram or chart such as the adapted chart in the PRISMA-ScR. Studies will be excluded if full-text documents cannot be retrieved. In addition, articles will also be excluded if they focus on culture in intra- or interprofessional encounters, describe culture in terms of art (dance, painting, music, etc.) or are published as editorials, debates, calls to action, commentaries, or similar texts. Excluded publications and reasons for exclusion will also be reported in an appendix for all publications examined in full text.

Data extraction:

For peer review only

Data from the included full-text publications will be processed using the Covidence software and exported to a Microsoft Excel spreadsheet containing the following headings: Author(s), Year of publication, where the source was published or conducted (country of origin), aims/purpose, population and sample size, methodology/methods, outcomes and details of these (if applicable), conceptualization of culture as well as key findings that relate to the scoping review questions. If needed during the charting process, the data extraction spreadsheet might be updated to include additional headings to better capture content relevant to the scoping review. Extraction will be conducted by EW and JS. Callibration of extraction will be conducted by 5-10 articles being independently processed by both reviewers. Discrepancies in extraction will be discussed until negotiated consensus is reached and the extraction form will be adjusted accordingly if needed.

Data analysis and presentation:

Data analysis will consist of summarizing the extracted data related to methodology, professions, and geographical contexts to answer related research questions. In addition, extracted data related to the conceptualization of culture and key findings will be analyzed using qualitative content analysis and discourse analysis. The results will be described in relation to the research questions and as appropriate in relation to each analysis methodology. The results of the discourse analysis of the included publications will be presented in a separate publication.

Ethics and dissemination

This review does not require ethical approval. The dissemination strategy includes peer review publication and presentation at conferences and to relevant stakeholders.

Author contributions:

EW and JS conceptualized the idea and study design. SLS and CL contributed to discussions on study design, conducted searches of existing literature reviews and conducted initial test searches to clarify search terms and search strategies. EW drafted the first version of the protocol. JS, SLS and CL critically commented and/or revised the text.

Furthermore, all authors have given final approval of the version to be published and agree to be accountable for all aspects of the work.

Funding statement:

This research received no specific grant from any funding agency in the public, commercial or not-for-profit sectors.

Competing interests statement:

The authors declare that they have no competing interests.

For peer review only

Word Count: 2 665 words.

For peer review only

References:

1. McAuliffe M, Triandafyllidou A eds. World Migration Report 2022. Geneva: International Organization for Migration (IOM) 2021.
2. World Health Organization. World report on the health of refugees and migrants. 2022. https://www.who.int/teams/health-and-migration-programme/world-report-on-the-health-of-refugees-and-migrants
3. Ladha R, Neiterman E. Shades of care: Understanding the needs of racially and ethnically diverse paediatric patients, their families, and health care providers in North America. Journal of child health care 2021; 13674935211041863. doi:10.1177/13674935211041863
4. Radjack R, Touhami F, Woestelandt L, et al. Cultural Competence of Professionals Working With Unaccompanied Minors: Addressing Empathy by a Shared Narrative. Front. Psychiatry 2020;11. doi: 10.3389/fpsyt.2020.00528
5. Zambri F, Marchetti F, Colaceci S, et al. Taking care of minor migrants' health: The professionals' perception and training needs. Annali dell'Istituto superiore di sanita 2020;56:470–477. doi:10.4415/ANN_20_04_09
6. Olcoń K, Gulbas LE. "Because That's the Culture": Providers' Perspectives on the Mental Health of Latino Immigrant Youth. Qualitative Health Research 2018;28(12):1944–1954. doi:10.1177/1049732318795674
7. Stier J. The blindspots and biases of intercultural communication studies: A discussion on episteme and doxa in a field. Intercultural Communication 2010;24;1-9. http://www.immi.se/intercultural/nr24/stier-24.htm
8. Kagawa Singer M, Dressler W, George S, et al. Culture: The missing link in health research. Social science & medicine 2016;170:237–246. doi:10.1016/j.socscimed.2016.07.015
9. Baltag V, Pachyna A, Hall J. Global Overview of School Health Services: Data from 102 Countries. Health Behavior & Policy Review 2015;2(4):268-283. doi:10.14485/HBPR.2.4.4
10. Sveriges kommuner och regioner. Nuläge och utmaningar i elevhälsan 2021. https://skr.se/skr/tjanster/rapporterochskrifter/publikationer/nulageochutmaningarielevhalsan2021.66776.html
11. Michaud P-A, Vervoort JPM, Visser A, et al. Organization and activities of school health services among EU countries. European Journal of Public Health 2021;1-6. doi:10.1093/eurpub/ckaa200
12. Beck KF, Hämäläinen J. Mapping the field of international comparative research in school social work. International Social Work 2022;65(2):203–223. doi:10.1177/0020872819897742
13. Jimerson SR, Skokut M, Cardenas S, et al. Where in the World is School Psychology?: Examining Evidence of School Psychology Around the Globe. School Psychology International 2008;29(2):131-144. doi:10.1177/0143034308090056
14. Farrell P, Jimerson SR, Kalambouka A, et al. Teachers' Perceptions of School Psychologists in Different Countries. School Psychology International 2005;26(5):525-544. doi:10.1177/0143034305060787
15. Stier J, Sandström M. "Managing the unmanageable: curriculum challenges and teacher strategies in multicultural preschools in Sweden". Journal of Intercultural Communication 2018;48.
16. Musliu E, Vasic S, Clausson EK, et al. School nurses' experiences working with unaccompanied refugee children and adolescents: A qualitative study. SAGE Open Nursing 2019;5:1-8. doi:10.1177/2377960819843713
17. Inkeroinen S, Hupli M, Stolt M. School nurses' experiences of health promotion for school-age asylum seekers. The journal of school nursing 2020;1059840520978197. doi:10.1177/1059840520978197
18. Vega D, Lasser J, Plotts C. Global migration: The need for culturally competent school psychologists. School Psychology International 2015;36(4):358-374. doi:10.1177/0143034315587011
19. Villarreal Sosa L, Roth B, Rodriguez S. Crossing Borders: Exploring the Role of School Social Workers in Immigrant-Serving Schools, Social Work Research 2021;45(3):198–206. doi:10.1093/swr/svab011
20. Wahlström E, Golsäter M, Granlund M, et al. Adjusting and doing the same: School nurses' descriptions of promoting participation in health visits with children of foreign origin. BMC Public Health 2021;21(111):1-11. doi:10.1186/s12889-020-10144-2
21. Villarreal Sosa L, Martin M. Constructions of Race and Equity in a Suburban School: Teachers, School Social Workers, and Other School Staff as Nepantleras and Border Crossers. Children & Schools 2021;43(1):19–31. doi:10.1093/cs/cdaa031
22. Peters MDJ, Godfrey C, McInerney P, et al. Best practice guidance and reporting items for the development of scoping review protocols. JBI evidence synthesis 2022;20(4):953–968. doi:10.11124/JBIES-21-00242
23. Peters MDJ, Godfrey C, McInerney P, et al. Chapter 11: Scoping Reviews (2020 version). In: Aromataris E, Munn Z, eds. JBI Manual for Evidence Synthesis, JBI, 2020. Available from https://synthesismanual.jbi.global. doi:10.46658/JBIMES-20-12
24. Khalil H, Peters MDJ, Tricco AC, et al. Conducting high quality scoping reviews-challenges and solutions. Journal of clinical epidemiology 2021;130:156–160. doi:10.1016/j.jclinepi.2020.10.009
25. Mak S, Thomas A. Steps for Conducting a Scoping Review. Journal of graduate medical education 2022;14(5):565–567. doi:10.4300/JGME-D-22-00621.1

For peer review only - http://bmjopen.bmj.com/site/about/guidelines.xhtml

Preferred Reporting Items for Systematic reviews and Meta-Analyses extension for Scoping Reviews (PRISMA-ScR) Checklist

SECTION	ITEM	PRISMA-ScR CHECKLIST ITEM	REPORTED ON PAGE #
**TITLE**			
Title	1	Identify the report as a scoping review.	1
**ABSTRACT**			
Structured summary	2	Provide a structured summary that includes (as applicable): background, objectives, eligibility criteria, sources of evidence, charting methods, results, and conclusions that relate to the review questions and objectives.	1-2
**INTRODUCTION**			
Rationale	3	Describe the rationale for the review in the context of what is already known. Explain why the review questions/objectives lend themselves to a scoping review approach.	3-4
Objectives	4	Provide an explicit statement of the questions and objectives being addressed with reference to their key elements (e.g., population or participants, concepts, and context) or other relevant key elements used to conceptualize the review questions and/or objectives.	4
**METHODS**			
Protocol and registration	5	Indicate whether a review protocol exists; state if and where it can be accessed (e.g., a Web address); and if available, provide registration information, including the registration number.	This is the protocol
Eligibility criteria	6	Specify characteristics of the sources of evidence used as eligibility criteria (e.g., years considered, language, and publication status), and provide a rationale.	5
Information sources*	7	Describe all information sources in the search (e.g., databases with dates of coverage and contact with authors to identify additional sources), as well as the date the most recent search was executed.	6
Search	8	Present the full electronic search strategy for at least 1 database, including any limits used, such that it could be repeated.	6
Selection of sources of evidence†	9	State the process for selecting sources of evidence (i.e., screening and eligibility) included in the scoping review.	7
Data charting process‡	10	Describe the methods of charting data from the included sources of evidence (e.g., calibrated forms or forms that have been tested by the team before their use, and whether data charting was done independently or in duplicate) and any processes for obtaining and confirming data from investigators.	7
Data items	11	List and define all variables for which data were sought and any assumptions and simplifications made.	5-6
Critical appraisal of individual sources of evidence§	12	If done, provide a rationale for conducting a critical appraisal of included sources of evidence; describe the methods used and how this information was used in any data synthesis (if appropriate).	No quality appraisal is conducted

For peer review only

SECTION	ITEM	PRISMA-ScR CHECKLIST ITEM	REPORTED ON PAGE #
Synthesis of results	13	Describe the methods of handling and summarizing the data that were charted.	7
**RESULTS**			
Selection of sources of evidence	14	Give numbers of sources of evidence screened, assessed for eligibility, and included in the review, with reasons for exclusions at each stage, ideally using a flow diagram.	Inapplicable
Characteristics of sources of evidence	15	For each source of evidence, present characteristics for which data were charted and provide the citations.	Inapplicable
Critical appraisal within sources of evidence	16	If done, present data on critical appraisal of included sources of evidence (see item 12).	Inapplicable
Results of individual sources of evidence	17	For each included source of evidence, present the relevant data that were charted that relate to the review questions and objectives.	Inapplicable
Synthesis of results	18	Summarize and/or present the charting results as they relate to the review questions and objectives.	Inapplicable
**DISCUSSION**			
Summary of evidence	19	Summarize the main results (including an overview of concepts, themes, and types of evidence available), link to the review questions and objectives, and consider the relevance to key groups.	Inapplicable
Limitations	20	Discuss the limitations of the scoping review process.	Inapplicable
Conclusions	21	Provide a general interpretation of the results with respect to the review questions and objectives, as well as potential implications and/or next steps.	7
**FUNDING**			
Funding	22	Describe sources of funding for the included sources of evidence, as well as sources of funding for the scoping review. Describe the role of the funders of the scoping review.	8

JBI = Joanna Briggs Institute; PRISMA-ScR = Preferred Reporting Items for Systematic reviews and Meta-Analyses extension for Scoping Reviews.
* Where *sources of evidence* (see second footnote) are compiled from, such as bibliographic databases, social media platforms, and Web sites.
† A more inclusive/heterogeneous term used to account for the different types of evidence or data sources (e.g., quantitative and/or qualitative research, expert opinion, and policy documents) that may be eligible in a scoping review as opposed to only studies. This is not to be confused with *information sources* (see first footnote).
‡ The frameworks by Arksey and O'Malley (6) and Levac and colleagues (7) and the JBI guidance (4, 5) refer to the process of data extraction in a scoping review as data charting.
§ The process of systematically examining research evidence to assess its validity, results, and relevance before using it to inform a decision. This term is used for items 12 and 19 instead of "risk of bias" (which is more applicable to systematic reviews of interventions) to include and acknowledge the various sources of evidence that may be used in a scoping review (e.g., quantitative and/or qualitative research, expert opinion, and policy document).

From: Tricco AC, Lillie E, Zarin W, O'Brien KK, Colquhoun H, Levac D, et al. PRISMA Extension for Scoping Reviews (PRISMAScR): Checklist and Explanation. Ann Intern Med. 2018;169:467–473. doi: 10.7326/M18-0850.

For peer review only - http://bmjopen.bmj.com/site/about/guidelines.xhtml
